# Quantitative Proteomic Analysis Reveals Changes in the Benchmark *Corynebacterium pseudotuberculosis* Biovar *Equi* Exoproteome after Passage in a Murine Host

**DOI:** 10.3389/fcimb.2017.00325

**Published:** 2017-07-25

**Authors:** Wanderson M. Silva, Rodrigo D. De Oliveira Carvalho, Fernanda A. Dorella, Edson L. Folador, Gustavo H. M. F. Souza, Adriano M. C. Pimenta, Henrique C. P. Figueiredo, Yves Le Loir, Artur Silva, Vasco Azevedo

**Affiliations:** ^1^Departamento de Biologia Geral, Instituto de Ciências Biológicas, Universidade Federal de Minas Gerais Belo Horizonte, Brazil; ^2^Institut National de la Recherche Agronomique (INRA), UMR1253 Science & Technologie du Lait & de l'Oeuf (STLO) Rennes, France; ^3^Agrocampus Ouest, UMR1253 Science & Technologie du Lait & de l'Oeuf (STLO) Rennes, France; ^4^Escola de Veterinária, Universidade Federal de Minas Gerais Belo Horizonte, Brazil; ^5^Centro de Biotecnologia, Universidade Federal da Paraíba João Pessoa, Brazil; ^6^Waters Corporation, Waters Technologies Brazil, MS Applications Laboratory São Paulo, Brazil; ^7^Departamento de Bioquímica e Imunologia, Instituto de Ciências Biológicas, Universidade Federal de Minas Gerais Belo Horizonte, Brazil; ^8^Instituto de Ciências Biológicas, Universidade Federal do Pará Belém, Brazil

**Keywords:** *Corynebacterium pseudotuberculosis*, label-free proteome, ulcerative lymphangitis, bacterial proteome, bacterial virulence, serial passage

## Abstract

*Corynebacterium pseudotuberculosis* biovar *equi* is the etiologic agent of ulcerative lymphangitis. To investigate proteins that could be related to the virulence of this pathogen, we combined an experimental passage process using a murine model and high-throughput proteomics with a mass spectrometry, data-independent acquisition (LC-MS^E^) approach to identify and quantify the proteins released into the supernatants of strain 258*_equi*. To our knowledge, this approach allowed characterization of the exoproteome of a *C. pseudotuberculosis equi* strain for the first time. Interestingly, the recovery of this strain from infected mouse spleens induced a change in its virulence potential, and it became more virulent in a second infection challenge. Proteomic screening performed from culture supernatant of the control and recovered conditions revealed 104 proteins that were differentially expressed between the two conditions. In this context, proteomic analysis of the recovered condition detected the induction of proteins involved in bacterial pathogenesis, mainly related to iron uptake. In addition, KEGG enrichment analysis showed that ABC transporters, bacterial secretion systems and protein export pathways were significantly altered in the recovered condition. These findings show that secretion and secreted proteins are key elements in the virulence and adaptation of *C. pseudotuberculosis*. Collectively, bacterial pathogenesis-related proteins were identified that contribute to the processes of adherence, intracellular growth and evasion of the immune system. Moreover, this study enhances our understanding of the factors that may influence the pathogenesis of *C. pseudotuberculosis*.

## Introduction

*Corynebacterium pseudotuberculosis* is a gram-positive, facultative intracellular pathogen that is globally distributed and can infect horses, cattle, sheep, goats, buffalos, and occasionally humans. *C. pseudotuberculosis* biovar *ovis* is the etiologic agent of caseous lymphadenitis in small ruminants (Dorella et al., 2006). *Corynebacterium pseudotuberculosis* strains belonging to biovar *equi*, however, cause edematous skin illness in buffalos. In horses, the infection can manifest through one of two forms: (i) externally, which usually presents with chronic ventral and pectoral lymph node abscesses and, in a more advanced stage, generates an illness denominated ulcerative lymphangitis that is characterized by ulcers of irregular shapes and sizes, or (ii) internally, which is characterized mainly by abscess formation in the lymph nodes and internal organs (kidney, liver, lung, and spleen) (Britz et al., 2014). In the United States, ulcerative lymphangitis outbreaks with large economic losses for horse farmers have been reported (Aleman et al., 1996; Foley et al., 2004; Spier, 2008). In addition, a recent study showed an increase in the case numbers of horses infected with *C. pseudotuberculosis* biovar *equi* during the last 10 years in the western region of the USA, which is being considered as an endemic area (Kilcoyne et al., 2014).

Whole genome sequencing of *C. pseudotuberculosis* biovar e*qui* strain 258, isolated from a horse with ulcerative lymphangitis in Belgium, revealed the presence of pathogenic islands in its chromosome as well as genes that might contribute to its virulence, most of them coding for secreted proteins. Moreover, putative antigenic proteins were identified through reverse vaccinology (Soares et al., 2013a). Some studies have shown that extracellular proteins are related to the pathogenic process of *C. pseudotuberculosis* (Wilson et al., 1995; Billington et al., 2002; Pacheco et al., 2012; Seyffert et al., 2014). However, phospholipase D (Pld) exotoxin, which contributes to bacterial spread in the host, is considered the major virulence factor of this pathogen (McKean et al., 2007). In addition, some secreted factors related to the virulence of *C. pseudotuberculosis* have already been described, such as serine protease CP40 (Wilson et al., 1995) and two operons, *fagABC* and *ciuABCDEF*, that are involved in iron uptake (Billington et al., 2002; Ribeiro et al., 2014).

The study of host-bacteria interactions in natural hosts, such as horses, cattle or sheep, is difficult because of the underlying genetic variability among animals; it is also extremely expense and requires multiple replicates and control animals. Thus, several studies have used mice as a model for studying both the pathogenic process (Jolly, 1965; Zaki, 1966; Nieto et al., 2009) and vaccination testing against infection by *C. pseudotuberculosis* (Simmons et al., 1997; Lan et al., 1999; Gorman et al., 2010; Ribeiro et al., 2014; Droppa-Almeida et al., 2016). In regards to host-bacteria interactions, some work has explored the serial passage process of bacterial pathogens *in vitro* or in an *in vivo* model to identify factors that might be involved in virulence (Fernández et al., 2000, 2013; Bleich et al., 2005; Chapuis et al., 2011; Fernandez-Brando et al., 2012; Liu et al., 2015). In this study, we adopted an *in vivo* assay in which the strain 258_*equi* was experimentally inoculated in mice followed by high-throughput proteomic analysis. We screened the functional genome of 258_*equi* after experimental passage in the murine host by examining the proteins released into the culture supernatant of this strain using the three-phase partitioning (TPP) protocol for obtaining extracellular proteins (Paule et al., 2004) and a mass spectrometry, data-independent acquisition (LC-MS^E^) approach to identify and quantify the proteins (Silva et al., 2006; Pacheco et al., 2011).

## Materials and methods

### Bacterial strain and growth conditions

*Corynebacterium pseudotuberculosis* biovar *equi* strain 258 was isolated from a horse in Belgium; this strain was cultivated under routine conditions in brain–heart infusion broth (BHI-HiMedia Laboratories Pvt. Ltd., India) at 37°C. When necessary, 1.5% agar was added to the medium for solid culture. For extracellular proteomic analyses, 258*_equi* was grown in a chemically defined medium (CDM) [(Na_2_HPO_4__7H_2_O (12.93 g/L), KH_2_PO_4_ (2.55 g/L), NH_4_Cl (1 g/L), MgSO_4__7H_2_O (0.20 g/L), CaCl_2_ (0.02 g/L), and 0.05% (v/v) Tween 80] with 4% (v/v) MEM Vitamins Solution (Invitrogen, Gaithersburg, MD, USA), 1% (v/v) MEM Amino Acids Solution (Invitrogen), 1% (v/v) MEM Non-Essential Amino Acids Solution (Invitrogen), and 1.2% (w/v) glucose at 37°C (Moura-Costa et al., 2002).

### Experimental infection in a murine model

The infection parameters were performed according to Moraes et al. (2014) and Ribeiro et al. (2014). In this study, female BALB/c mice between 6- and 8-weeks-old were utilized; they were provided by the Animal Care Facility at the Biological Sciences Institute at the Federal University of Minas Gerais and were handled in accordance with the CEUA guidelines of the UFMG Ethics Committee on Animal Testing (Permit Number: CETEA 103/2011). For the bacterial passage assay, three mice were infected via intraperitoneal injection with 10^6^ colony forming units (CFU) of strain 258_*equi*. Thirty-six hours after infection, the animals were sacrificed, and the spleen was aseptically removed for recovering the bacteria. Each spleen was individually macerated in a sterile saline solution (0.9% NaCl_2_) and seeded onto BHI agar plates for incubation at 37°C for 48 h. Subsequently, one bacterial colony of each BHI plate was isolated and cultured in BHI broth at 37°C with shaking (180 rpm) until the OD_600_ = 0.8. Three different stock cultures were generated and stored at −80°C in BHI broth and 10% glycerol. The recovered bacteria are referred to as Recovered (Rc), and bacteria with no previous host contact were used as the Control (Ct). For bacterial virulence assays, bacteria from the three individual frozen stocks of Rc and the Ct condition were centrifuged at 5,000 × g for 5 min and washed twice in saline solution, followed by resuspension in saline solution. Three groups of five mice were infected with bacteria from the Rc or Ct condition via intraperitoneal injection of a suspension containing 10^6^ or 10^5^ CFU. The animals' survival rates were calculated and represented in GraphPad Prism v.5.0 (GraphPad Software, San Diego, CA, USA) using the Kaplan-Meier survival function.

### Preparation of extracellular proteins for proteome analysis

For proteomic analysis, three independent control and recovered colonies from the three individual frozen stocks were grown in CDM to an OD600 = 0.8. The cultures were then centrifuged for 20 min at 2,700 × g. The supernatants were filtered using 0.22-μm filters, 30% (w/v) ammonium sulfate was added to the samples, and the pH of the mixtures was adjusted to 4.0. Next, 20 mL/L N-butanol was added to each sample. The samples were centrifuged for 10 min at 1,350 × g and 4°C. The interfacial precipitate was collected and resuspended in 1 mL of 20 mM Tris-HCl, pH 7.2 (Paule et al., 2004). Proteins were quantified using the Bradford assay. For label-free proteomic analysis, the protein extract was concentrated using a spin column with a 10 kDa threshold (Millipore, Billerica, MA, USA). The protein was denatured (0.1% *Rapi*GEST SF at 60°C for 15 min) (Waters, Milford, CA, USA), reduced (10 mM DTT), alkylated (10 mM iodoacetamide) and enzymatically digested with trypsin (Promega, Sequencing Grade Modified Trypsin, Madison, WI, USA). Glycogen phosphorylase (Waters Corporation, SwissProt P00489) was added to the digests to a final concentration of 20 fmol/μl as an internal standard for normalization prior to each replicate injection. The digestion process was stopped by adding 10 μL of 5% TFA (Fluka, Buchs, Germany) (Silva et al., 2006).

### Mass spectrometry analysis, data processing and quantification

Three independent biological replicates of each experimental condition were digested, as described above, for MS^E^ analysis. Qualitative and quantitative nanoUPLC tandem nanoESI-HDMS^E^ (Nano Electrospray High Definition Mass Spectrometry) experiments were performed using a 1 h reversed-phase gradient from 7 to 40% (v/v) acetonitrile (with 0.1% v/v formic acid) at 500 nL.min^−1^ using a nanoACQUITY UPLC 2D RPxRP Technology system (Gilar et al., 2005). All analyses were performed using nano-electrospray ionization in the positive ion mode (nanoESI (+)) and a NanoLockSpray (Waters, Manchester, UK) ionization source. The mass spectrometer was calibrated with an MS/MS spectrum of human [Glu1]-Fibrinopeptide B (Glu-Fib) solution (100 fmol.mL−1) delivered through the reference sprayer of the NanoLockSpray source. The double-charged ion ([M + 2H]^2+^ = 785.8426) was used for initial single-point calibration, and MS/MS fragment ions of Glu-Fib were used to obtain the final instrument calibration. Multiplexed data-independent (DIA) scanning with additional specificity and selectivity for non-linear “T-wave” ion mobility (HDMS^E^) experiments were performed using a Synapt G2-S HDMS mass spectrometer (Waters, Manchester, UK), which was constructed to automatically switch between the application of standard MS (3 eV) and elevated collision energies HDMS^E^ (19–45 eV) to the transfer “T-wave” CID (collision-induced dissociation) cell with argon gas.

The proteins were identified, and quantitative data were packaged using dedicated algorithms (Silva et al., 2005; Geromanos et al., 2009) and searching against a database with default parameters to account for ions (Li et al., 2009). The databases used were reversed “on-the-fly” during the database queries and appended to the original database to assess the false positive rate during identification. For proper spectra processing and database searching conditions, the ProteinLynx Global SERVER v.2.5.2 (PLGS) with Identity^E^ and Expression^E^ informatics v.2.5.2 (Waters, Manchester, UK) was used. UniProtKB (release 2013_01) with manually reviewed annotations was used, and the search conditions were based on taxonomy (*Corynebacterium pseudotuberculosis*). The maximum allowed missed cleavages by trypsin was up to 1, and various modifications, including carbamidomethyl (C), N-terminal acetyl, phosphoryl (STY) and oxidation (M), were allowed. A peptide mass tolerance value of 10 ppm was used. The search threshold to accept each spectrum was the default value in the program with a false discovery rate value of 4% (Curty et al., 2014). For protein quantitation, PLGS v2.5.2 software was used with the Identity^E^ algorithm using Hi3 methodology and glycogen phosphorylase (muscle form; P00489) peptides were used as internal standards. The collected proteins were organized by the PLGS Expression^E^ tool algorithm into a statistically significant list (*p*-value ≤ 0.05) that corresponded to higher or lower regulation ratios between the different groups. The calculation of the log ratio and the confidence interval was based on a Gaussian distribution model, which allows for the possibility of an uncertain peptide assignment, an incorrect assignment of data to a cluster or interference. The confidence interval of 95% was used, and the probability distribution of the measured value of a log2 ratio more than a 1.2 was more symmetric than that obtained for the direct ratio, making the results interpretations more meaningful (Levin et al., 2011). For comparing pairs of experimental groups, proteins with a differential expression log_2_ ratio greater than or equal to 1.2 between the two conditions were considered for higher or lower abundance level determination (Levin et al., 2011).

### Bioinformatics analysis

The proteins identified in both conditions were analyzed using the following prediction tools: SurfG+ v1.0 (Barinov et al., 2009) was used to predict subcellular localization, SecretomeP 2.0 server was used to predict proteins exported from non-classical systems (positive prediction scores greater than 0.5; Bendtsen et al., 2005a), TatP was used to predict proteins with twin-arginine signal peptides (Bendtsen et al., 2005b) and the PIPs software was used to predict the proteins in pathogenicity islands (Soares et al., 2012). Gene ontology (GO) functional annotations were generated using the COG data base (Tatusov et al., 2001). Pathway enrichment analysis of significant proteins was carried out using the Kyoto encyclopedia of genes and genomes (KEGG) database. A protein-protein interaction network was generated using Cytoscape version 2.8.3 (Shannon et al., 2003) with a spring-embedded layout.

## Results

### Evaluation of the virulence potential of 258_*equi* after passage in a murine host

**Figure 1:**
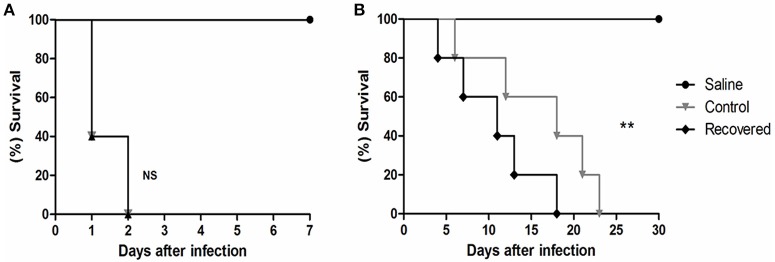
Survival assay of Balb/C mice infected with strain 258_*equi*. **(A)** Percent survival of BALB/c mice infected with 10^6^ CFU of bacteria. **(B)** Percent survival of BALB/c mice infected with 10^5^ CFU of bacteria. Ct, control condition and Rc, recovered condition. The mortality rates were measured daily. The results presented in **(A,B)** represents three independent experiments. The *p-*values were calculated using the log rank test. Note that infection with 10^5^ CFU of bacteria changes the potential virulence of Rc (*p* = 0.0024, log-rank test) relative to Ct. NS = *P* > 0.05; ^**^P < 0.01.

In the first *in vivo* assay, BALB/c mice were infected with 10^6^ CFU of bacteria that had no previous host contact and with bacteria that were recovered from mouse spleens. We observed that all of the infected animals under both the control (Ct) and recovered (Rc) conditions died within 48 h of infection (Figure 1A). These results reveal the virulence potential of 258_*equi*; however, in this assay, we did not observe differences in the virulence potential between the control and recovered condition. Next, the Ct and Rc condition were analyzed using a new survival assay in BALB/c mice, but a 10^5^ CFU infection dose was used (Figure 1B). In this assay, we observed altered virulence in the Rc condition; the mice began dying in the first 10 days post-infection (40% decrease in survival rate) and mortality reached 100% in less than 20 days post-infection. For the Ct condition, while mice died during the first 10 days of infection, this early stage only resulted in 20% mortality, and mortality did not reach 100% until 23 days post-infection. Finally, we detected abscess formation in the internal organs (kidney and liver) of all animals infected with either the Ct or Rc condition in the assays using 10^5^ CFU (data not shown). These results show that passage in a murine host affects the virulence potential of 258_*equi*.

### Overview of the exoproteome of *C. pseudotuberculosis* strain 258 after passage in a murine host

After passage of 258_*equi* in BALB/c mice, we detected changes in its virulence potential. To assess whether this change is reflected in its proteome, considering the importance of exported proteins in bacterial infection (Hilbi et al., 2012), we used a TPP/LC-MS^E^ approach (Pacheco et al., 2011) to compare the extracellular proteome of the control and recovered conditions. From our proteomic analysis, a total of 113 non-redundant 258_*equi* proteins were detected with high confidence and were identified in at least two of the three biological replicates of the two conditions tested, with an average of 17 peptides per protein and an FDR of 1%. The peptides were identified with a normal distribution of 10 ppm error for the total identified peptides (Supplementary Figure 1A). In addition, only the source fragments of peptides with a charge state of at least [M + 2H]^2+^ and the absence of decoys were considered to increase data quality.

The absolute quantitation of proteins present within a complex protein mixture is extremely important for understating physiological adaptations in response to biological demands promoted by environmental changes (Mallick and Kuster, 2010). To estimate the absolute abundance of identified proteins in the 258_*equi* exoproteome, we utilized the Hi3 method (Silva et al., 2006) where the average MS signal responses for the three most intense tryptic peptides for each protein were determined, including those of the internal standard protein glycogen phosphorylase (muscle form; P00489). All samples were normalized prior to injection using “scouting runs,” and the stoichiometry between the intensity and molarity proportion prior to the replicate runs per condition were considered. From this analysis a dynamic range of protein abundance was generated spanning three orders of magnitude (Supplementary Figure 1B). Lysozyme M1 was the most abundant protein detected. This protein, which is related to bacterial virulence, is localized in the pathogenic island Cp258PiCp02. Lysozyme M1 was also detected in a membrane shaving of a field isolate of *C. pseudotuberculosis* biovar *ovis* (Rees et al., 2015). Other proteins, such as hydrolase domain containing protein, trehalose corynomycolyl transferase B, which is involved in the cell wall synthesis, and FtsX, a protein related to division cellular, were among the most abundant proteins. All of the identified proteins on the protein abundance scale are listed in Supplementary Table 1.

For evaluating the relative differences between the core exoproteome of the Ct and Rc conditions, we used label-free quantification (Silva et al., 2005, 2014; Pacheco et al., 2011). In agreement with the PLGS analyses, 105 proteins between the Rc and Ct conditions presented significant statistical values (*p* < 0.05) using the Expression^E^ algorithm tool (Supplementary Table 2). Differential expression was considered for proteins that were significantly different (*p* < 0.05) and had log_2_ ratios equal to or greater than a factor of 1.2, as described by Levin et al. (2011). Based on this analysis, 39 proteins were induced and 16 were down-regulated in the Rc condition (Table 1). In addition, we detected proteins exclusive to the proteome of each condition; cytochrome c nitrate reductase small subunit NrfH was detected only in the Ct condition. In only the Rc condition, two multidrug resistance proteins, the cytochrome oxidase assembly, a thioredoxin-related protein and three proteins with unknown function were identified (Supplementary Table 3).

**Table 1:** Proteins differentially expressed between the recovered (Rc) and control (Ct) conditions.

**Accession**	**Description**	**Score**	**Gene**	**Rc:Ct Log(2)Ratio**
**ADHESION AND MOTILITY CELL**				
I3QZX5_CORPS	Sortase A	13261.7	*srtA*	1.28
**AMINO ACID TRANSPORT AND METABOLISM**				
I3QWW3_CORPS	Diaminopimelate decarboxylase	600.78	*lysA*	−1.27
I3QXT1_CORPS	Chorismate synthase aroC	614.35	*aroC*	−1.54
I3QY54_CORPS	4-hydroxy-tetrahydrodipicolinate reductase	1212.64	*dapB*	−1.67
**CELL DIVISION AND CELLULAR CYCLE**				
I3QW64_CORPS	Cell division protein FtsX	2967.84	*ftsX*	1.88
I3QYI0_CORPS	Cell division protein FtsQ	4225.31	*ftsQ*	1.24
I3QYH4_CORPS	Antigen 84	31337.36	*ag84*	1.23
**CELL WALL/MEMBRANE AND ENVELOPE BIOGENESIS**				
I3R031_CORPS	Trehalose corynomycolyl transferase B	112067.8	*cmtB*	3.10
I3QV43_CORPS	Penicillin binding protein transpeptidase	18512.68	*pbpB*	1.80
I3QZM5_CORPS	D-alanyl-D-alanine carboxypeptidase	2988.16	*pbp4*	1.46
I3QX04_CORPS	Mycothiol acetyltransferase	5934.73	*mshD*	−4.62
**COENZYME METABOLISM**				
I3QVB7_CORPS	Uroporphyrinogen decarboxylase	3376.83	*hemE*	1.89
**DNA METABOLISM: REPLICATION, RECOMBINATION AND REPAIR**				
I3QXT3_CORPS	Amino deoxychorismate lyase	4162.99	*yceG*	1.56
**GENERAL FUNCTION PREDICTION ONLY**				
I3QZJ3_CORPS	Lipoprotein LpqE	44732.01	*lpqE*	3.74
I3QXX7_CORPS	Lipoprotein	36815.57	*Cp258_1221*	3.62
I3QXE1_CORPS	Hemolysin related protein	893.83	*tlyC*	1.75
I3QXC3_CORPS	Esterase	496.62	*Cp258_1017*	1.30
I3QZ50_CORPS	Peptidase S8A Subtilisin family	40174.72	*Cp258_1653*	1.23
I3QW71_CORPS	Periplasmic binding protein	11884.29	*fecB*	1.21
I3QW24_CORPS	Hydrolase domain containing protein	17234.12	*Cp258_0564*	−1.26
I3QYP5_CORPS	MutT NUDIX family protein	5870.55	*Cp258_1498*	−1.38
I3QV42_CORPS	Protein yqeY	23153.72	*yqeY*	−1.57
I3R0F7_CORPS	Anthranilate synthase component II	382.53	*trpG*	−1.63
I3QXJ1_CORPS	Prolipoprotein LppL	2671.28	*lppL*	−3.38
I3QZA3_CORPS	Protein NrdI	7211.97	*nrdI*	−4.70
**INORGANIC ION TRANSPORT AND METABOLISM**				
I3QVU3_CORPS	Cell surface hemin receptor HtaA	11874.87	*htaA*	2.22
I3QUS8_CORPS	Iron-regulated membrane protein	7389.79	*piuB*	1.85
I3QUW4_CORPS	ABC type metal ion transport system	1016.6	*mntA*	1.75
I3QXC5_CORPS	CiuA protein	12242.99	*ciuA*	1.64
I3QVU4_CORPS	Hemin binding periplasmic protein HmuT	14010	*hmuT*	1.47
I3QVU6_CORPS	Hemin import ATP binding protein HmuV	785.22	*hmuV*	1.34
I3QUM8_CORPS	FagC protein	392.95	*fagC*	1.23
I3QX10_CORPS	Iron(3+)-hydroxamate-binding protein FhuD	13922.33	*fhuD*	1.21
I3QUW5_CORPS	Manganese zinc iron transport system ATP-binding	391.52	*mntB*	−1.38
**INTRACELLULAR TRAFFICKING, SECRETION, AND VESICULAR TRANSPORT**				
I3QX59_CORPS	ABC transporter domain containing protein	1150.79	*Cp258_0956*	1.79
I3QXV8_CORPS	Protein translocase subunit SecF	1729.78	*secF*	1.66
I3R0D7_CORPS	Oligopeptide binding protein OppA	3469.47	*oppA7*	1.50
I3QWP1_CORPS	Oligopeptide binding protein OppA	41781.98	*oppA3*	1.38
I3QZC0_CORPS	ABC type antimicrobial peptide transport	1159.51	*Cp258_1740*	1.34
I3QXV9_CORPS	Protein translocase subunit SecD	2879.45	*secD*	1.24
**LIPID TRANSPORT AND METABOLISM**				
I3QW96_CORPS	Enoyl CoA hydratase echA6	611.75	*echA6*	−1.40
**POST-TRANSLATIONAL MODIFICATION, PROTEIN TURNOVER, CHAPERONES**				
I3QW38_CORPS	Lon protease	6439.26	*lon*	1.34
**UNKNOWN FUNCTION**				
I3QYD5_CORPS	Unknown Function	8143.98	*Cp258_1380*	4.17
I3QZP8_CORPS	Unknown Function	14676.76	*Cp258_1869*	2.93
I3QW25_CORPS	Unknown Function	1433.27	*Cp258_0565*	2.74
I3R0E2_CORPS	Unknown Function	141172.41	*Cp258_2121*	2.60
I3QW83_CORPS	Unknown Function	1589.85	*Cp258_0622*	2.39
I3QWP8_CORPS	Unknown Function	54353.63	*Cp258_0793*	2.29
I3R049_CORPS	Unknown Function	2318.32	*Cp258_2028*	2.11
I3QZK0_CORPS	Unknown Function	1030.59	*Cp258_1819*	1.37
I3QV90_CORPS	Unknown Function	12638.51	*Cp258_0263*	1.34
I3QVZ1_CORPS	Unknown Function	2256.66	*Cp258_0531*	−1.28
I3QYV3_CORPS	Unknown Function	141.46	*Cp258_1555*	−1.53
I3QWK1_CORPS	Unknown Function	221.77	*Cp258_0745*	−2.02
I3R080_CORPS	Unknown Function	2564.2	*Cp258_2060*	−2.64

### *In silico* prediction of 258_*equi* exoprotein localization

**Figure 2:**
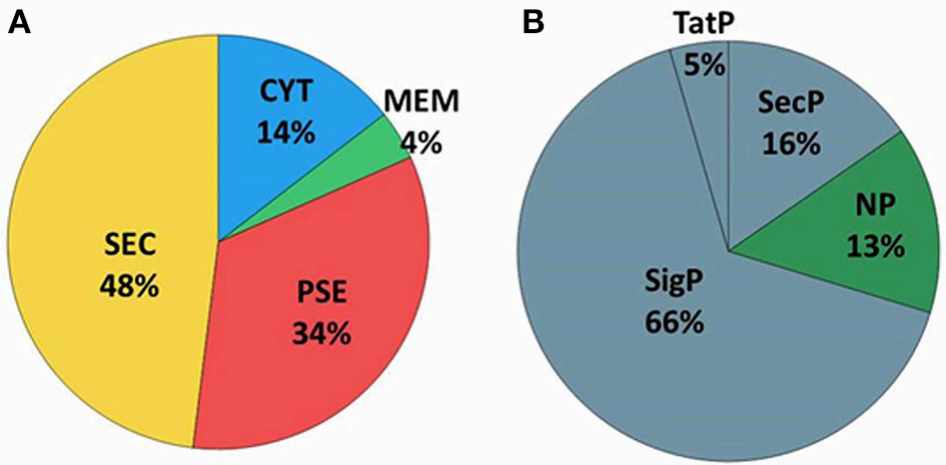
Prediction of the subcellular localization of the 258_*equi* exoproteome. **(A)** CYT, cytoplasmic; MEM, membrane; PSE, potentially surface-exposed; and SEC, secreted. **(B)** SecP, SecretomeP prediction for non-classical pathways; TatP, Tat-pathway prediction; SigP, SignalP prediction for peptide signal prediction; and NP, no prediction for SecP, TatP, or SigP.

Extracellular proteins produced by prokaryotic organisms are a subset of proteins present in the extracellular milieu, which is composed of both proteins with signal peptides that are actively secreted by classical secretion systems and proteins without signal sequences that are exported by non-classical secretion systems (Bendtsen et al., 2005a; Desvaux et al., 2009). To identify proteins that contain signal peptides and to determine their subcellular localizations, we utilized the SurfG tool (Barinov et al., 2009), which enables the classification of proteins within the following categories: cytoplasmic (CYT), membrane (MEM), potentially surface-exposed (PSE) and secreted (SEC) (Figure 2A and Supplementary Tables 2, 3). Of the total proteins identified, 66% (*n* = 74) presented positive predictions for signal peptides (Figure 2B). This group was composed of predicted proteins in the SEC and PSE categories. When these results were compared with the *in silico* data of the 258_*equi* genome, we had identified approximately 65% and 16% of the proteins predicted to be SEC and PSE, respectively. The proteins that did not present positive predictions for signal peptides were analyzed by SecretomeP (Bendtsen et al., 2005a) to identify proteins that could eventually be exported by non-classical secretion systems. According this analysis, nine proteins were predicted to be PSE, seven proteins were predicted to be MEM and two proteins were predicted to be CYT as they presented High SecP scores above 0.5 (Supplementary Tables 2, 3), suggesting that these proteins might be exported by a non-classical secretion system. Taken together, 86% of the 258_*equi* exoproteome was composed of extracellular proteins (Figure 2B). In addition, our proteomic analysis detected 17 proteins predicted to be lipoproteins (Supplementary Table 2).

### Functional classifications of the differentially expressed proteins in strain 258_*equi* after passage in a murine host

**Figure 3:**
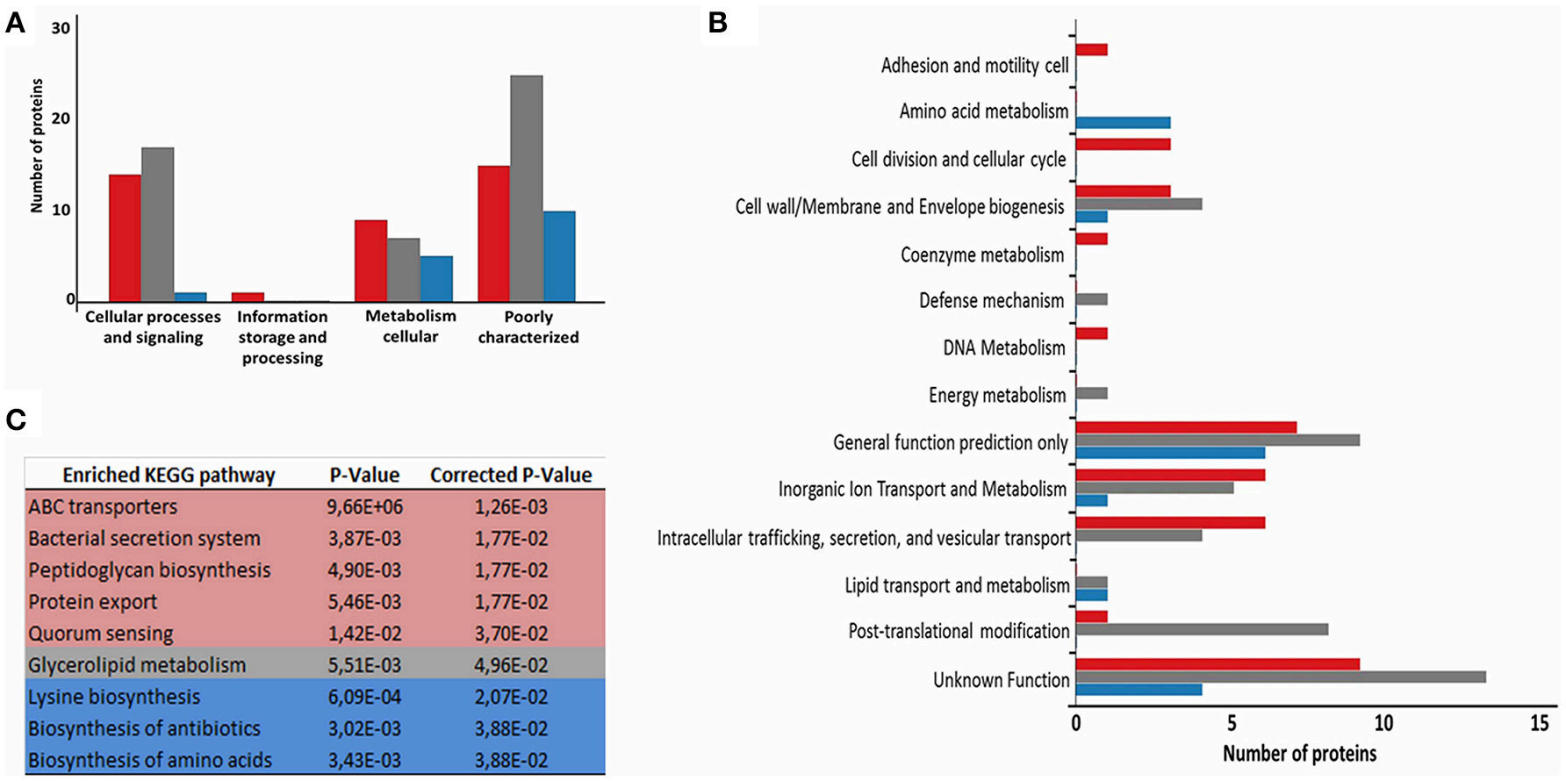
Functional analysis of the differentially expressed proteome between the control and recovered conditions. **(A)** Proteins classified by COG functional categories. **(B)** Categorization of differentially expressed proteins in biological processes. **(C)** KEGG pathway enrichment analysis of differentially expressed proteins. The colors are based on the Rc:Ct relation; red, up-regulated; gray, unchanged; and blue, down-regulated.

To evaluate the functional characteristics of the 258_*equi* exoproteome, we performed a Clusters of Orthologous Groups analysis (Tatusov et al., 2001). According GO analysis, the proteins were organized by clusters of orthologous groups (Figure 3A). When we evaluated each functional category, we observed that the majority of the proteins detected as induced in the Rc condition were predicted as “function unknown” and “general function only” (Table 1 and Figure 3B). These results represent a lack of knowledge regarding a protein set that might play an important role in the pathogenic process of 258_*equi*, and therefore, more studies are necessary to investigate the true roles of these proteins in the virulence of this strain. When we evaluated proteins with known or predicted functions, the majority of those that were more abundant in the Rc condition were related to cellular processes and signaling (Figures 3A,B). According to *in silico* data of the 258_*equi* genome, this pathogen has five iron uptake systems (Supplementary Figure 2; Soares et al., 2013a). Interestingly, in our proteomic analysis, we identified components of each of the 5 systems as more abundant in the Rc condition, including CiuA, FhuD, FagC, HmuT, HmuV, and HtaA (Table 1), suggesting that iron uptake pathways may play an important role in the pathogenesis of *C. pseudotuberculosis*. Moreover, we detected several proteins related to bacterial pathogenesis that contribute to processes of adherence, intracellular growth and evasion of the immune system (Table 1 and Supplementary Tables 2, 3).

**Figure 4:**
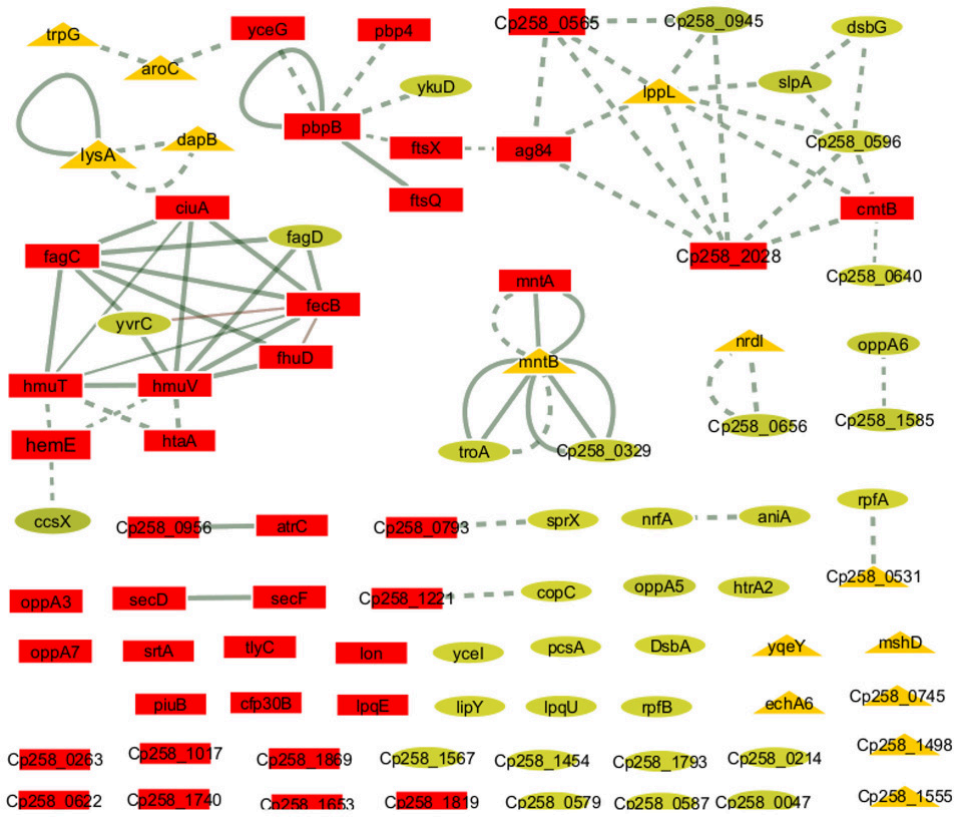
Protein-protein interaction network. The network nodes represent proteins, and the edges represent protein-protein associations. The node size (protein) is proportional to the amount of protein interacting (degree of interaction). Dotted line = regulatory interactions (functional), solid line, physical interactions; triangles, down-regulated proteins in the Rc condition; squares, highly induced proteins in the Rc condition; and circles, unchanged proteins.

To identify the most relevant biological pathways of the proteins differentially expressed between the Ct and Rc conditions, we performed a KEGG enrichment analysis. Enrichment results revealed eight biological pathways with significant differences (*p* < 0.05). The proteins that were induced in the Rc condition are in pathways such as ABC transporters, bacterial secretion systems, peptidoglycan biosynthesis and protein export (Figure 3C). This finding confirms that secretion and secreted proteins are key elements in *C. pseudotuberculosis* virulence and adaptation, as suggested by previous reports that identified several secreted proteins as potential virulence factors in *C. pseudotuberculosis* (Pacheco et al., 2011; Silva et al., 2013; Rees et al., 2015). Most proteins perform their function in a context of networks by interacting with other proteins (Schleker et al., 2012). To evaluate the 258_*equi* exoproteome at the network level, we performed a protein-protein interaction analysis of the differentially expressed proteins using the Cytoscape tool. After Cytoscape analysis, the 258_*equi* exoproteome network was composed of 87 proteins (Figure 4). In the PPI-network, we observed enrichment clusters in heme biosynthesis and ABC transporters related to iron uptake, peptidoglycan biosynthesis and antibiotic biosynthesis. In addition, we observed that some clusters were formed by unknown proteins, which shows that these proteins may play an important role in the virulence of *C. pseudotuberculosis*.

## Discussion

Here, we report a comprehensive analysis of the exoproteome of an *equi* isolate of *C. pseudotuberculosis*. The 258_*equi* exoproteome was composed of a high number of extracellular proteins, and a similar result was observed in a study conducted by Pacheco et al. (2011), which characterized the extracellular proteomes of *C. pseudotuberculosis* biovar *ovis* strains. The infections caused by *C. pseudotuberculosis* are chronic in character, and due to this, post-infection disease signs may not begin to appear until after 6 months. Necropsy is the only viable way to identify abscesses, but the cost is high. Testing several strains requires many animals and would result in high economic and ethical costs. Thus, mice are used as an alternative model for studying *C. pseudotuberculosis* infection, because they are relatively resistant to experimental challenge and are able to contain infection (Jolly, 1965; Zaki, 1966). In addition, mice have been shown to be efficient for the evaluation of different vaccine compounds and of humoral and cellular immune responses (Simmons et al., 1997; Lan et al., 1999; Gorman et al., 2010; Droppa-Almeida et al., 2016). Other studies have used mice to study virulence and pathogenesis, including an evaluation of hepatic disease (Nieto et al., 2009), or to study knockout strains (Moraes et al., 2014; Ribeiro et al., 2014).

In our study, the serial passage process in mice was efficient to induce changed both virulence and functional genome of 258_*equi*, which was showed through of proteomic analysis. In the *in vivo* assay, we observed changes in its virulence potential in a new infection assay with a lower infection dose. Similar results from an evaluation of the virulence potential of Shiga toxin (Stx)-producing *Escherichia coli* (STEC) were also observed when mice were infected with a lower dose of the recovered bacteria that had been recovered after serial passage in a murine model (Fernandez-Brando et al., 2012). Other studies have also shown that the serial passage process through *in vitro* or *in vivo* models leads to changes in the virulence potential of pathogens, including *Helicobacter pylori, Escherichia coli, Xenorhabdus nematophila, Arcobacter butzleri, Salmonella enterica*, and *Shigella flexneri* (Fernández et al., 2000, 2013; Bleich et al., 2005; Chapuis et al., 2011; Fernandez-Brando et al., 2012; Liu et al., 2015). Changes in the virulence potential of these pathogens, as well as in 258_*equi*, show that this strategy promotes the activation of genes related to bacterial pathogenesis.

**Figure 5:**
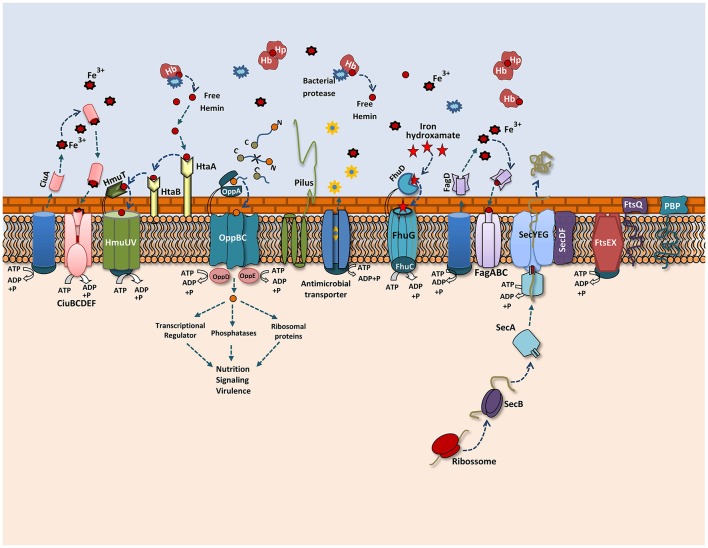
Overview of the 258_*equi* proteome after the recuperation process. A model representing the main exoproteins induced in the recovered condition, including proteins related to biogenesis of the cell wall, cellular adhesion and different secretion pathways related to iron acquisition, bacterial nutrition, efflux pumps and the Sec pathway.

A proteomic study conducted with *Shigella flexneri* after passage in an *in vivo* model showed the induction of important proteins that might contribute to its adaptation process during infection (Liu et al., 2015). In our proteomic analysis, we also observed changes in the 258_*equi* exoproteome after the recuperation process, and proteins that might play an important role in the pathogenesis of *C. pseudotuberculosis* were detected (Figure 5). Interestingly, when compared the proteins that were differentially induced in the Rc condition, with *in silico* data of the core-genome of *C. pseudotuberculosis* biovar *equi* and biovar *ovis* strains (Soares et al., 2013b), we observed that all proteins are present in this core-genome. This result represents a set of proteins that might be important to pathogenesis of biovar *equi* and biovar *ovis* strains. Within our proteomic repertoire, we detected predicted proteins such as lipoprotein. This class of proteins is produced by several prokaryotic organisms and then translocated across the membrane through the Sec or Tat pathway (Pugsley, 1993; Shruthi et al., 2010). Different studies have shown that these peripherally anchored membrane proteins perform an important role in the physiology, virulence and immune response of different gram-negative and gram-positive pathogens. In addition, lipoproteins are recognized as excellent vaccine targets (Nguyen and Götz, 2016). In the closely related pathogen *M. tuberculosis*, lipoproteins have been shown to be extremely important for virulence, contributing directly to evasion of the immune system (Su et al., 2016).

Adhesion to host cells is a key determinant that contributes to bacteria–host interaction; this process is required for bacterial colonization and persistence. *In vitro* and *in silico* studies showed that *C. pseudotuberculosis* contains pili, and these structures play an important role in cellular adhesion (Yanagawa and Honda, 1976; Soares et al., 2013b). In *C. pseudotuberculosis, spaA* is a major pili gene that is encoded by the following gene cluster: *srtB*-*spaA*-*srtA*-*spaB*-*spaX*-*spaC* (Soares et al., 2013b). We found that sortase A (SrtA) was induced in the Rc supernatant. This cell surface anchored transpeptidase catalyzes the covalent attachment of precursor cell wall-attached proteins (LPXTG proteins) to the peptidoglycan. In gram-positive pathogens, such as *Listeria monocytogenes* (Bierne et al., 2002), *Streptococcus pneumoniae* (Kharat and Tomasz, 2003), and *Staphylococcus aureus* (Oh et al., 2006), *srtA* mutant strains had reduced virulence in animal infection models. We also detected important proteins related to the cell division and growth of *Corynebacterium* induced in the Rc condition, such as FtsQ and FtsX, which form part of the *ftsXE* cluster, and penicillin-binding proteins (PBPs) (Letek et al., 2008). In *E. coli*, the FtsEX proteins were suggested to form an ABC transporter system involved in the uptake of substrates necessary to maintain osmotic pressure during cell division (Schmidt et al., 2004; Reddy, 2007). FtsX was detected among the most abundant proteins of the 258_*equi* exoproteome, which suggests it is an important protein within the biology of this strain. PBPs proteins have an important role in cell-wall biosynthesis in *Corynebacterium* as they are essential to peptidoglycan biosynthesis. In addition, this class of proteins is a target of antibiotics (Letek et al., 2008). Antigen 84 (Ag84) was also induced in the Rc condition. Interestingly, Ag84 was also detected in a membrane shaving of an *ovis* strain isolated directly from the caseous nodes of a diseased animal (Rees et al., 2015). In *M. tuberculosis*, this protein presents antigenic characteristics and is required for growth (Sassetti et al., 2003). These proteins may have key functions in the replication and growth of *C. pseudotuberculosis* during the infection process.

Iron is an essential element for both the virulence and growth of several bacterial pathogens during the infection process. However, free iron is not available to the bacterial inside the host, thus several pathogens utilize different mechanisms to acquire both free iron and iron from host iron proteins (Brown and Holden, 2002). For *C. pseudotuberculosis*, iron acquisition is a required step in its pathogenic process (Billington et al., 2002; Ribeiro et al., 2014), and according to an *in silico* analysis of the 258_*equi* genome, this bacterium has different genetic loci associated with high-affinity iron transport systems as well as surface-associated heme-uptake pathways (Soares et al., 2012). In our proteomic analysis, we detected that specific proteins related to iron acquisition were induced in the Rc condition. Some these proteins are involved directly in the virulence of *C. pseudotuberculosis*, such as the FagC protein that is component of the Fag system. A study performed with strains with defective *fagB(C)* genes showed that these strains presented reduced virulence in goats (Billington et al., 2002).

To acquire iron inside a host, bacteria synthesize and secrete siderophores, which are low-molecular-weight iron chelators that have a high and specific affinity for ferric iron (Ellermann and Arthur, 2017). We detected the CiuA siderophore, which is localized in the operon *ciuABCDE* (*Corynebacterium* iron uptake). Studies performed with the pathogen *C. diphtheria* showed that Ciu is a high-affinity iron uptake system (Kunkle and Schmitt, 2005). Additionally, in a study performed with *C. pseudotuberculosis*, a *ciuA* mutated strain showed reduced virulence, demonstrating the role of this protein in the virulence of this bacterium, and was also able to protect immunized mice when they were challenged with a virulent strain (Ribeiro et al., 2014). Another siderophore detected was the FhuD siderophore, which is part of the conserved ferric hydroxamate uptake system, Fhu. The uptake of ferric ferrichrome is described in pathogens such as *L. monocytogenes* (Jin et al., 2006; Xiao et al., 2011), *Streptococcus pyogenes* (Hanks et al., 2005) and *S. aureus* (Sebulsky and Heinrichs, 2001). In *L. monocytogenes*, FhuD contributes to the uptake of ferric hydroxamate from ferrichrome, ferrichrome A and ferrioxamine B (Jin et al., 2006; Xiao et al., 2011). In *S. aureus*, this protein was shown to contribute both to proliferation within the blood and to the formation of renal abscesses in mice (Mishra et al., 2012). Like *C. diphtheria*, the genome of 258_*equi* also has genetic loci with genes related to heme acquisition, such as the hemin-uptake (hmu) operon *HmuTUV* and cell hemin specific receptors *htaA, htaB* and *htaC*. The ABC hemin transporter HmuTUV, together with cell-surface hemin receptors, is involved in heme uptake from hemoglobin (Hb), hemoglobin/haptoglobin, and myoglobin (Mb) (Kunkle and Schmitt, 2005; Allen and Schmitt, 2015). The presence of these systems shows the versatility of 258_*equi* in acquiring iron from different sources. Interestingly, the HtaA protein was detected to be immunoreactive in an immunoproteomic study of *C. pseudotuberculosis* biovar *ovis* (Seyffert et al., 2014). Taken together, these results indicate that, similar to other pathogens, iron acquisition likely plays an important role in 258_*equi* virulence as this strain uses distinct iron acquisition systems during infection. This ability to acquire iron may contribute to the increase virulence observed in the 258*_equi* strain.

Opp transport systems belong to the superfamily of conserved ATP-binding cassette transporters and play an important role in bacterial nutrition, signaling and virulence (Yu et al., 2014). The OppA protein, which is responsible for the uptake of peptides from the external medium, was induced in the Rc supernatant. In *Mycobacterium avium*, the *oppA* gene contributed to infections in a mouse model as well as to its viability in macrophages (Danelishvili et al., 2014). The Sec pathway is the major secretion system in several prokaryotic pathogens, components of this system was also induced in the Rc supernatant. SecDF are accessory factors from this translocation machinery and act to increase protein translocation. Different studies show that in *S. aureus*, the role of SecDF is related to the export of several virulence factors that contribute to parts its pathogenic process, such as adhesion, invasion and immune system evasion (Sibbald et al., 2006). In addition, SecDF belong to the resistance-nodulation-cell division (RND) family of multidrug export pumps and contribute to the resistance process against the antimicrobial effects of cathelicidins, a class of antimicrobial peptides produced by the immune system (Blodkamp et al., 2017). Similarly, proteins for antimicrobial agent resistance, such as ABC-type antimicrobial peptide transporters, which are localized in the pathogenicity island Cp258PiCp14, and efflux transporters, such as NorM, which belongs to the multidrug and toxic compound extrusion (MATE) transporter, were also detected. These data are consistent with previous *in vitro* studies, which showed that *C. pseudotuberculosis* is resistant to several classes of antimicrobial agents (Judson and Songer, 1991), and that activation of these defense pathways against antimicrobial agents might contribute to survival of this pathogen.

## Conclusion

Herein, we characterized, for the first time, the exoproteome of a *C. pseudotuberculosis equi* isolate. In addition, we showed changes in both the virulence and proteomic profiles of 258*_equi* after its recovery from murine host spleens. Through a TPP/LC-MS^E^ approach, we detected secreted virulence-associated proteins. The up-regulation of these proteins may account for the difference in virulence potential we observed in the Rc condition compared with the Ct condition. Altogether, our proteomic repertoire identified several extracellular proteins involved in key processes of bacterial pathogenesis that might contribute to the pathogenic process of *C. pseudotuberculosis*.

## Author contributions

WS, VA, and YL Conceived and designed the experiments. WS and FD performed *in vivo* experiments. WS and RD performed microbiological analyses and sample preparation for proteomic analysis. WS and GS conducted the proteomic analysis. EF performed bioinformatics analysis. AP, YL, and HF contributed substantially to data interpretation and revisions. AS, VA, and YL participated in all steps of the project as coordinators, and critically reviewed the manuscript.

### Conflict of interest statement

The authors declare that the research was conducted in the absence of any commercial or financial relationships that could be construed as a potential conflict of interest.

## Supplementary material

Supplementary Figure 1 **(A)** Two-dimensional nanoUPLC HDMS^E^ analysis showing the distribution of fragment masses and the exact mass accuracy for 90% of the precursor ions with a 10 ppm maximum error. **(B)** Dynamic range based on the absolute quantitation of the proteins identified by LC-HDMS^E^ analysis.

Supplementary Figure 2 Genomic loci encoding proteins related to iron-acquisition in 258_*equi*. Red genes encode proteins that were identified in our proteomic analysis.

Supplementary Table 1 Total list of identified proteins on the protein abundance scale.

Supplementary Table 2 Total list of differentially expressed proteins between the recovered and control conditions of stain 258*_equi*.

Supplementary Table 3 Proteins unique to the recovered and control conditions.
