# Supplementary figures and images for: Quantitative Proteomic Analysis Reveals Changes in the Benchmark Corynebacterium pseudotuberculosis Biovar Equi Exoproteome after Passage in a Murine Host
Source: Front Cell Infect Microbiol. 2017 Jul 25;7:325. doi: 10.3389/fcimb.2017.00325 (PMC5524672; doi:10.3389/fcimb.2017.00325)

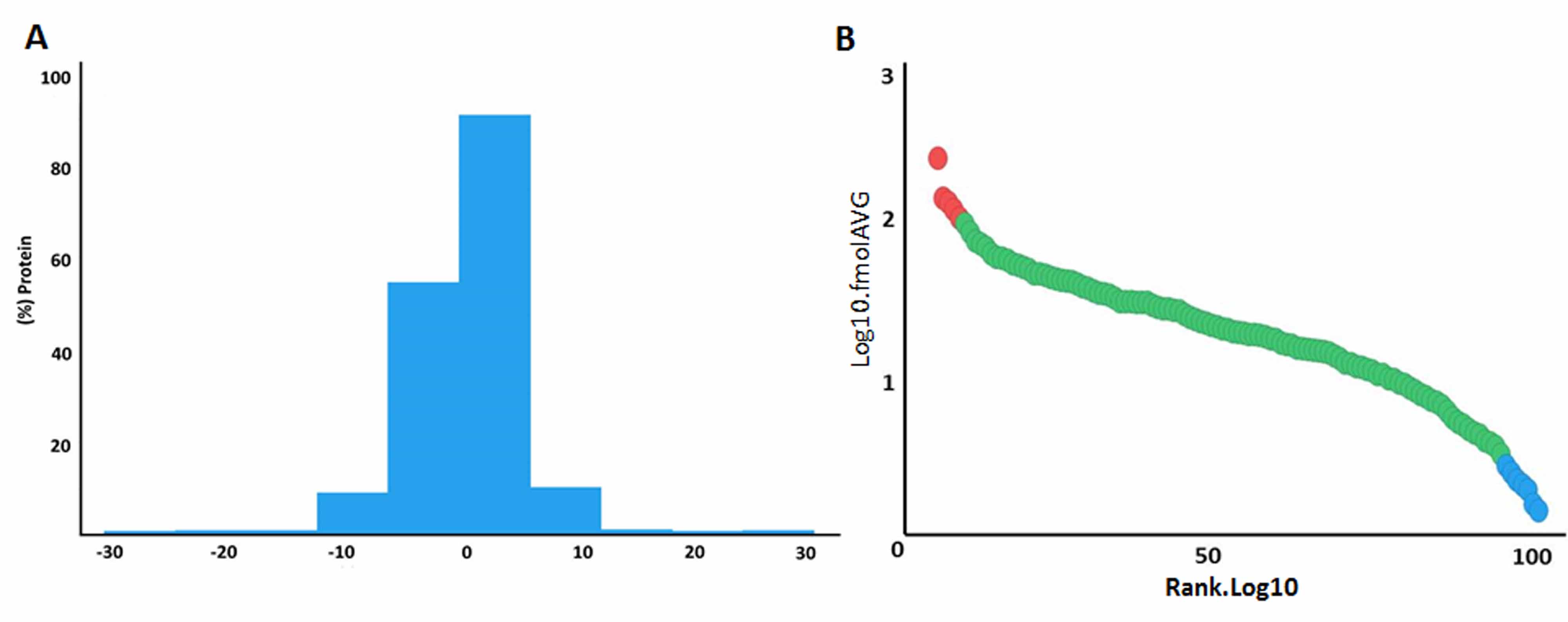

Supplement: Supplementary Figure 1 — (A) Two-dimensional nanoUPLC HDMSE analysis showing the distribution of fragment masses and the exact mass accuracy for 90% of the precursor ions with a 10 ppm maximum error. (B) Dynamic range based on the absolute quantitation of the proteins identified by LC-HDMSE analysis. [file Image1.JPEG]

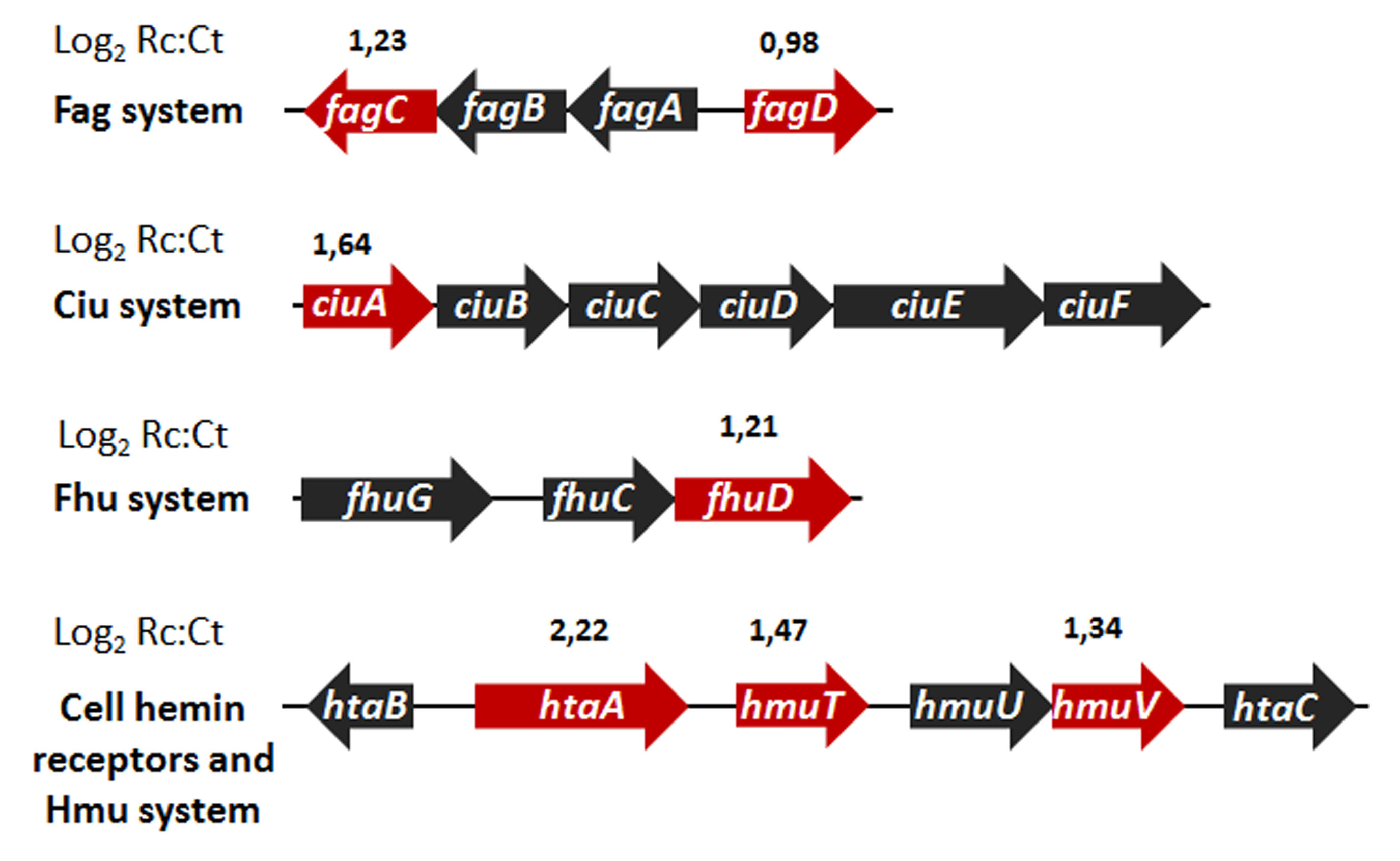

Supplement: Supplementary Figure 2 — Genomic loci encoding proteins related to iron-acquisition in 258_equi. Red genes encode proteins that were identified in our proteomic analysis. [file Image2.JPEG]
