# Supplementary material for: Quantitative Proteomic Analysis Reveals Changes in the Benchmark Corynebacterium pseudotuberculosis Biovar Equi Exoproteome after Passage in a Murine Host
Source: Front Cell Infect Microbiol. 2017 Jul 25;7:325. doi: 10.3389/fcimb.2017.00325 (PMC5524672; doi:10.3389/fcimb.2017.00325)
Supplement: Supplementary Table 1 — Total list of identified proteins on the protein abundance scale. [file Table1.PDF]

Supplementary Table 1: Total list of identified proteins on the protein abundance scale.

protein.Accession	protein.Description	Log10FmolAVG	rankByLog10
I3QUS1_CORPS	Lysozyme M1	2.415010486	1
I3QZS9_CORPS	Uncharacterized protein	2.337653033	2
I3QW24_CORPS	Hydrolase domain containing protein	2.140079681	3
I3QW64_CORPS	Cell division protein FtsX	2.106977041	4
I3R031_CORPS	Trehalose corynomycolyl transferase B	2.060762919	5
I3R0E2_CORPS	Uncharacterized protein	2.021687727	6
I3QX38_CORPS	Cutinase	1.961692655	7
I3QV79_CORPS	Surface layer protein A	1.902364846	8
I3QUM7_CORPS	Phospholipase D	1.844433851	9
I3QW80_CORPS	Resuscitation promoting factor	1.824217821	10
I3QW71_CORPS	Periplasmic binding protein	1.81578713	11
I3QWP8_CORPS	Uncharacterized protein	1.792873653	12
I3QW83_CORPS	Uncharacterized protein	1.730821463	13
I3QWW3_CORPS	Diaminopimelate decarboxylase	1.722996985	14
I3QV40_CORPS	Uncharacterized protein	1.711410509	15
I3QYI0_CORPS	Cell division protein FtsQ	1.684652663	16
I3QVU3_CORPS	Cell surface hemin receptor	1.672494569	17
I3QWP5_CORPS	Trypsin like serine protease	1.660400126	18
I3QYP0_CORPS	Cell surface hemin receptor	1.644770763	19
I3QWG4_CORPS	Resuscitation promoting factor RpfB	1.614568845	20
I3QYP5_CORPS	MutT NUDIX family protein	1.614210839	21
I3QWJ5_CORPS	LpqU family protein	1.607947624	22
I3QUN1_CORPS	Iron siderophore binding protein	1.588480488	23
I3QX49_CORPS	Uncharacterized protein	1.582083868	24
I3QXX8_CORPS	Copper resistance protein CopC	1.569203999	25
I3QZP8_CORPS	Uncharacterized protein	1.564002281	26
I3QZC0_CORPS	ABC type antimicrobial peptide transport	1.562652381	27
I3QWA0_CORPS	Uncharacterized protein	1.546902816	28
I3QZJ3_CORPS	Lipoprotein LpqE	1.546649055	29
I3QW96_CORPS	Enoyl CoA hydratase echA6	1.525998004	30
I3QZ55_CORPS	L-D-transpeptidase YkuD	1.51768233	31
I3QXC5_CORPS	Iron ABC transporter substrate binding	1.495937173	32
I3QV43_CORPS	Penicillin binding protein transpeptidase	1.482451671	33
I3QXE1_CORPS	Hemolysin related protein	1.47717479	34
I3QVC2_CORPS	Thiol disulfide isomerase thioredoxin	1.468819457	35
I3QZ73_CORPS	Uncharacterized protein	1.43676499	36
I3QW38_CORPS	Lon protease	1.422621675	37
I3QUN5_CORPS	Penicillin binding protein A	1.420883482	38
I3QUN8_CORPS	FHA domain containing protein	1.420255464	39
I3QZH4_CORPS	Corynomycolyl transferase	1.419568583	40
I3QV73_CORPS	Hydrolase domain containing protein	1.416235	41

I3QUS8_CORPS	Putative iron regulated membrane protein	1.399903823	42
I3QUR9_CORPS	Uncharacterized protein	1.381418773	43
I3QZY0_CORPS	Oligopeptide binding protein oppA	1.374064492	44
I3QW45_CORPS	Copper containing nitrite reductase	1.372734323	45
I3QVA7_CORPS	Thiol disulfide interchange protein DsbG	1.361611201	46
I3QYG8_CORPS	Uncharacterized protein	1.360411108	47
I3QVU4_CORPS	Hemin binding periplasmic protein HmuT	1.335156569	48
I3QV90_CORPS	Uncharacterized protein	1.332483528	49
I3QZS6_CORPS	Cytochrome c nitrate reductase small	1.330657235	50
I3QVU6_CORPS	Hemin import ATP binding protein HmuV	1.317033673	51
I3QX10_CORPS	Iron 3 hydroxamate binding protein FhuD	1.262311506	52
I3QYV3_CORPS	Uncharacterized protein	1.25251037	53
I3QXJ1_CORPS	Prolipoprotein LppL	1.23735342	54
I3QZM5_CORPS	D alanyl D alanine carboxypeptidase	1.229462428	55
I3QZX5_CORPS	Fimbrial associated sortase like protein	1.215227167	56
I3QWK1_CORPS	Uncharacterized protein	1.213237082	57
I3QZM9_CORPS	Secretory lipase	1.205536532	58
I3QXV8_CORPS	Protein translocase subunit SecF	1.198240548	59
I3QUW8_CORPS	Periplasmic zinc binding protein TroA	1.197817484	60
I3QZS6_CORPS	Cytochrome c nitrate reductase small	1.189722961	61
I3QX04_CORPS	Mycothiol acetyltransferase	1.189566672	62
I3QXV9_CORPS	Protein translocase subunit SecD	1.167615038	63
I3QUU5_CORPS	Antigen Cfp30B	1.162602725	64
I3QWP1_CORPS	Oligopeptide binding protein oppA	1.137864244	65
I3QYK8_CORPS	Cell wall peptidase NlpC P60 protein	1.127715117	66
I3QVF5_CORPS	ABC type metal ion transport system	1.121490375	67
I3QVF4_CORPS	Uncharacterized protein	1.100412288	68
I3QX17_CORPS	Uncharacterized protein	1.096038986	69
I3QZ50_CORPS	Peptidase S8A Subtilisin family protein	1.091771494	70
I3QXX7_CORPS	Lipoprotein	1.085826549	71
I3R0D7_CORPS	Oligopeptide binding protein OppA	1.083016573	72
I3QYH4_CORPS	Antigen 84 b	1.079099256	73
I3R043_CORPS	ABC transporter substrate binding lipoprotein	1.068919838	74
I3R080_CORPS	Uncharacterized protein	1.067014859	75
I3QVZ1_CORPS	Uncharacterized protein	1.025098529	76
I3R0C1_CORPS	ABC 2 type transporter family protein	1.023708793	77
I3QZB4_CORPS	DsbG protein dot	0.991252469	78
I3QW47_CORPS	Uncharacterized protein	0.974653748	79
I3QUW5_CORPS	Manganese zinc iron transport system ATP	0.967796949	80
I3QZS5_CORPS	Nitrite reductase periplasmic cytochrome	0.953277224	81
I3QXT1_CORPS	Chorismate synthase ium	0.946095642	82
I3QVQ7_CORPS	Uncharacterized protein	0.919924939	83
I3QY54_CORPS	Dihydrodipicolinate reductase	0.919908178	84

I3QYX1_CORPS	ABC type dipeptide transport system	0.890346892	85
I3QYY1_CORPS	Uncharacterized protein	0.885691462	86
I3QZK0_CORPS	Uncharacterized protein	0.860244811	87
I3QWB6_CORPS	Glutaredoxin	0.799684173	88
I3QV53_CORPS	Trypsin like serine protease	0.787834499	89
I3QWA4_CORPS	Multidrug resistance protein NorM	0.765827406	90
I3QZA3_CORPS	Protein NrdI dot	0.740753463	91
I3QVD0_CORPS	Uncharacterized protein	0.669102918	92
I3QXC3_CORPS	Esterase	0.662957006	93
I3QY87_CORPS	Secreted penicillin binding protein	0.637404412	94
I3QW57_CORPS	Uncharacterized protein	0.634291939	95
I3QXN4_CORPS	Cytochrome oxidase assembly protein	0.582737412	96
I3R049_CORPS	Uncharacterized protein	0.566658066	97
I3QUW4_CORPS	ABC type metal ion transport system	0.558213487	98
I3QXT3_CORPS	Amino deoxychorismate lyase	0.531182762	99
I3QXF1_CORPS	Protein yceI	0.529316066	100
I3QV42_CORPS	Uncharacterized protein yqeY	0.525170386	101
I3QWR5_CORPS	Uncharacterized protein	0.520173391	102
I3QZ49_CORPS	Carbon starvation protein A	0.462119672	103
I3QYD5_CORPS	Uncharacterized protein	0.456433191	104
I3QV51_CORPS	Thioredoxin related protein eba	0.430373198	105
I3QX59_CORPS	ABC transporter domain containing protein	0.325864247	106
I3QYW5_CORPS	ABC transporter ATP binding protein	0.286063354	107
I3QW25_CORPS	Uncharacterized protein	0.230115077	108
I3QVB7_CORPS	Uroporphyrinogen decarboxylase	0.211301277	109
I3QY62_CORPS	Multidrug resistance protein NorM	0.120277728	110
I3QZU2_CORPS	Uncharacterized protein	0.098697731	111
I3R0F7_CORPS	Anthranilate synthase component II	0.021806548	112
I3QUM8_CORPS	ATP binding cytoplasmic membrane protein	-0.022997891	113
