# Supplementary material for: Quantitative Proteomic Analysis Reveals Changes in the Benchmark Corynebacterium pseudotuberculosis Biovar Equi Exoproteome after Passage in a Murine Host
Source: Front Cell Infect Microbiol. 2017 Jul 25;7:325. doi: 10.3389/fcimb.2017.00325 (PMC5524672; doi:10.3389/fcimb.2017.00325)
Supplement: Supplementary Table 2 — Total list of differentially expressed proteins between the recovered and control conditions of stain 258_equi. [file Table2.PDF]

Supplementary Table S.2. Total list of proteins differentially produced between the recovered and control conditions of stain 258_equi.

Accession	Description	Score	Log(2)Ratio[A]	Log(e)Ratio	Log(e)StdDev	(p)value[A]	SecP value	Subcellular localization	Locus_Tag	Lipoprotein	SignalP	TatP	Gene symbol	Biological Process	Biological Function	PIPs
I3QYD5_CORPS	Unknown Function	8143.98	4.17	2.89	0.42	1	0.895598	SEC	Cp258_1380		X		Cp258_1380	Unknown function	Poorly characterized	
I3QZJ3_CORPS	Lipoprotein LpqE	44732.01	3.74	2.59	0.12	1	0.908015	PSE	Cp258_1812	X	X		lpqE	General function prediction only	Poorly characterized	
I3QXX7_CORPS	Lipoprotein	36815.57	3.62	2.51	0.12	1	0.355013	PSE	Cp258_1221	X	X		Cp258_1221	General function prediction only	Poorly characterized	
I3R031_CORPS	Trehalose corynomycolyl transferase B	112067.8	3.1	2.15	0.07	1	0.228853	SEC	Cp258_2009		X		cmtB	Cell wall/Membrane and Envelope biogenesis	Cellular processes and signaling	
I3QZP8_CORPS	Unknown Function	14676.76	2.93	2.03	0.21	1	0.875428	SEC	Cp258_1869		X		Cp258_1869	Unknown function	Poorly characterized	
I3QW25_CORPS	Unknown Function	1433.27	2.74	1.9	0.28	1	0.505009	SEC	Cp258_0565		X		Cp258_0565	Unknown function	Poorly characterized	
I3R0E2_CORPS	Unknown Function	141172.41	2.6	1.8	0.05	1	0.953596	SEC	Cp258_2121		X		Cp258_2121	Unknown function	Poorly characterized	
I3QW83_CORPS	Unknown Function	1589.85	2.39	1.66	0.23	1	0.889508	SEC	Cp258_0622		X	X	Cp258_0622	Unknown function	Poorly characterized	
I3QWP8_CORPS	Unknown Function	54353.63	2.29	1.59	0.07	1	0.686841	SEC	Cp258_0793		X		Cp258_0793	Unknown function	Poorly characterized	
I3QVU3_CORPS	Cell surface hemin receptor HtaA	11874.87	2.22	1.54	0.12	1	0.946731	PSE	Cp258_0483		X		htaA	Inorganic Ion Transport and Metabolism	Metabolism cellular	
I3R049_CORPS	Unknown Function	2318.32	2.11	1.46	0.15	1	0.916746	SEC	Cp258_2028		X		Cp258_2028	Unknown function	Poorly characterized	
I3QVB7_CORPS	Uroporphyrinogen decarboxylase	3376.83	1.89	1.31	0.29	1	0.093524	SEC	Cp258_0292		X		hemE	Coenzyme metabolism	Metabolism cellular	
I3QW64_CORPS	Cell division protein FtsX	2967.84	1.88	1.3	0.15	1	0.840861	PSE	Cp258_0603				ftsX	Cell division and cellular cycle	Cellular processes and signaling	
I3QUS8_CORPS	Iron-regulated membrane protein	7389.79	1.85	1.28	0.19	1	0.867375	CYT	Cp258_0089				piuB	Inorganic Ion Transport and Metabolism	Metabolism cellular	
I3QV43_CORPS	Penicillin binding protein transpeptidase	18512.68	1.8	1.25	0.06	1	0.878622	SEC	Cp258_0217		X		pbpB	Cell wall/Membrane and Envelope biogenesis	Cellular processes and signaling	
I3QX59_CORPS	ABC transporter domain containing protein	1150.79	1.79	1.24	0.35	1	0.951661	MEM	Cp258_0956				Cp258_0956	Intracellular trafficking, secretion, and vesicular transport	Cellular processes and signaling	
I3QXE1_CORPS	Hemolysin related protein	893.83	1.75	1.21	0.31	1	0.777298	PSE	Cp258_1034				tlyC	General function prediction only	Poorly characterized	
I3QUW4_CORPS	ABC type metal ion transport system	1016.6	1.75	1.21	0.29	1	0.785750	SEC	Cp258_0126		X	X	mntA	Inorganic Ion Transport and Metabolism	Metabolism cellular	
I3QXV8_CORPS	Protein translocase subunit SecF	1729.78	1.66	1.15	0.14	1	0.926228	PSE	Cp258_1202				secF	Intracellular trafficking, secretion, and vesicular transport	Cellular processes and signaling	
I3QXC5_CORPS	Iron ABC transporter substrate binding CiuA	12242.99	1.64	1.14	0.07	1	0.939886	PSE	Cp258_1019	X	X		ciuA	Inorganic Ion Transport and Metabolism	Metabolism cellular	**>Cp258PiCp04**
I3QXT3_CORPS	Amino deoxychorismate lyase	4162.99	1.56	1.08	0.12	1	0.428064	SEC	Cp258_1177		X		yceG	DNA Metabolism: replication, recombination and repair	Information storage and processing	
I3R0D7_CORPS	Oligopeptide binding protein OppA	3469.47	1.5	1.04	0.24	1	0.861564	SEC	Cp258_2116		X		oppA7	Intracellular trafficking, secretion, and vesicular transport	Cellular processes and signaling	
I3QVU4_CORPS	Hemin binding periplasmic protein hmuT	14010	1.47	1.02	0.14	1	0.078207	PSE	Cp258_0484	X	X		hmuT	Inorganic Ion Transport and Metabolism	Metabolism cellular	
I3QZM5_CORPS	D-alanyl-D-alanine carboxypeptidase	2988.16	1.46	1.01	0.13	1	0.232261	SEC	Cp258_1844		X		pbp4	Cell wall/Membrane and Envelope biogenesis	Cellular processes and signaling	
I3QWP1_CORPS	Oligopeptide binding protein OppA	41781.98	1.38	0.96	0.06	1	0.879349	PSE	Cp258_0786		X		oppA3	Intracellular trafficking, secretion, and vesicular transport	Cellular processes and signaling	
I3QZK0_CORPS	Unknown Function	1030.59	1.37	0.95	0.46	1	0.943378	SEC	Cp258_1819		X		Cp258_1819	Unknown Function	Poorly characterized	
I3QVU6_CORPS	Hemin import ATP binding protein HmuV	785.22	1.34	0.93	0.22	1	0.042956	MEM	Cp258_0486				hmuV	Inorganic Ion Transport and Metabolism	Metabolism cellular	
I3QZC0_CORPS	ABC type antimicrobial peptide transport	1159.51	1.34	0.93	0.13	1	0.725407	PSE	Cp258_1740				Cp258_1740	Intracellular trafficking, secretion, and vesicular transport	Cellular processes and signaling	**>Cp258PiCp14**
I3QW38_CORPS	Lon protease	6439.26	1.34	0.93	0.15	1	0.547244	SEC	Cp258_0578		X		lon	Post-translational modification, protein turnover, chaperones	Cellular processes and signaling	
I3QV90_CORPS	Unknown Function	12638.51	1.34	0.93	0.16	1	0.875377	SEC	Cp258_0263		X		Cp258_0263	Unknown Function	Poorly characterized	
I3QXC3_CORPS	Esterase	496.62	1.3	0.9	0.28	1	0.869588	PSE	Cp258_1017				Cp258_1017	General function prediction only	Poorly characterized	
I3QZX5_CORPS	Sortase A	13261.7	1.28	0.89	0.12	1	0.563218	PSE	Cp258_1952		X		srtA	Adhesion and motility cell	Cellular processes and signaling	
I3QXV9_CORPS	Protein translocase subunit SecD	2879.45	1.24	0.86	0.09	1	0.885827	PSE	Cp258_1203		X		secD	Intracellular trafficking, secretion, and vesicular transport	Cellular processes and signaling	
I3QYI0_CORPS	Cell division protein FtsQ	4225.31	1.24	0.86	0.15	1	0.231034	SEC	Cp258_1426		X		ftsQ	Cell division and cellular cycle	Cellular processes and signaling	
I3QYH4_CORPS	Antigen 84	31337.36	1.23	0.85	0.1	1	0.101972	CYT	Cp258_1419				ag84	Cell division and cellular cycle	Cellular processes and signaling	
I3QUM8_CORPS	FagC	392.95	1.23	0.85	0.51	1	0.132922	PSE	Cp258_0037				fagC	Inorganic Ion Transport and Metabolism	Metabolism cellular	**>Cp258PiCp01**
I3QZ50_CORPS	Peptidase S8A Subtilisin family	40174.72	1.23	0.85	0.07	1	0.537955	SEC	Cp258_1653		X		Cp258_1653	General function prediction only	Poorly characterized	**>Cp258PiCp10**
I3QX10_CORPS	Iron(3+)-hydroxamate-binding protein FhuD	13922.33	1.21	0.84	0.11	1	0.851968	PSE	Cp258_0905	X	X		fhuD	Inorganic Ion Transport and Metabolism	Metabolism cellular	
I3QW71_CORPS	Periplasmic binding protein	11884.29	1.21	0.84	0.11	1	0.699065	PSE	Cp258_0610	X	X		fecB	General function prediction only	Poorly characterized	
I3QUN8_CORPS	FHA domain containing protein	5273.17	1.2	0.83	0.18	1	0.787766	SEC	Cp258_0047		X		Cp258_0047	General function prediction only	Poorly characterized	
I3QWP5_CORPS	Trypsin like serine protease	43808.27	1.17	0.81	0.05	1	0.715181	SEC	Cp258_0790		X		sprX	Post-translational modification, protein turnover, chaperones	Cellular processes and signaling	
I3QZS9_CORPS	Unknown Function	186862	1.17	0.81	0.04	1	0.345387	SEC	Cp258_1902		X		Cp258_1902	Unknown Function	Poorly characterized	
I3QUW8_CORPS	Periplasmic zinc binding protein TroA	4767.38	1.15	0.8	0.14	1	0.883516	SEC	Cp258_0130		X		troA	Inorganic Ion Transport and Metabolism	Metabolism cellular	
I3QX49_CORPS	Unknown Function	3140.93	1.13	0.78	0.15	1	0.745898	SEC	Cp258_0945		X		Cp258_0945	Unknown Function	Poorly characterized	
I3QUS1_CORPS	Lyzozyme M1	60294.48	1.1	0.76	0.03	1	0.566775	SEC	Cp258_0082		X		Cp258_0082	Cell wall/Membrane and Envelope biogenesis	Cellular processes and signaling	**>Cp258PiCp02**
I3QZU2_CORPS	Unknown Function	681.93	1.07	0.74	0.36	1	0.741836	SEC	Cp258_1917		X		Cp258_1917	Unknown Function	Poorly characterized	
I3QVC2_CORPS	Thiol disulfide isomerase thioredoxin	13524.61	1.04	0.72	0.23	1	0.584097	PSE	Cp258_0297	X	X		ccsX	Post-translational modification, protein turnover, chaperones	Cellular processes and signaling	
I3QWA0_CORPS	Unknown Function	30180.2	1.02	0.71	0.05	1	0.584278	SEC	Cp258_0640				Cp258_0640	Unknown function	Poorly characterized	
I3QYP0_CORPS	Cell surface hemin receptor	1201.96	1.00	0.69	0.17	1	0.921801	PSE	Cp258_1493		X		Cp258_1493	Inorganic Ion Transport and Metabolism	Metabolism cellular	**>Cp258PiCp05**
I3QZY0_CORPS	Oligopeptide binding protein oppA	12618	1.00	0.69	0.11	1	0.798788	SEC	Cp258_1957		X		oppA6	Intracellular trafficking, secretion, and vesicular transport	Cellular processes and signaling	**>Cp258PiCp07**
I3QVA7_CORPS	Thiol disulfide interchange protein DsbA	18489.59	1.00	0.69	0.1	1	0.552929	SEC	Cp258_0282		X		dsbA	Post-translational modification, protein turnover, chaperones	Cellular processes and signaling	
I3QUN1_CORPS	Iron siderophore binding protein FagD	24685.73	0.98	0.68	0.06	1	0.869174	SEC	Cp258_0040		X		fagD	Inorganic Ion Transport and Metabolism	Metabolism cellular	**>Cp258PiCp01**
I3QXF1_CORPS	Protein yceI	11630.7	0.98	0.68	0.13	1	0.909643	SEC	Cp258_1044		X		yceI	Unknown Function	Poorly characterized	
I3R0C1_CORPS	ABC 2 type transporter family protein	1156.96	0.97	0.67	0.27	1	0.839674	PSE	Cp258_2101				Cp258_2101	Intracellular trafficking, secretion, and vesicular transport	Cellular processes and signaling	
I3QWJ5_CORPS	LpqU family protein	796.04	0.97	0.67	0.28	1	0.480573	SEC	Cp258_0739		X		lpqU	General function prediction only	Poorly characterized	
I3QV53_CORPS	Trypsin like serine protease	786.84	0.95	0.66	0.34	1	0.555611	PSE	Cp258_0226				htrA2	Post-translational modification, protein turnover, chaperones	Cellular processes and signaling	
I3QUN5_CORPS	Penicillin binding protein A	7569.97	0.94	0.65	0.11	1	0.789009	SEC	Cp258_0044		X		pbpA	Cell wall/Membrane and Envelope biogenesis	Cellular processes and signaling	
I3QYK8_CORPS	Cell wall peptidase NlpC P60 protein	21880.81	0.92	0.64	0.08	1	0.715751	SEC	Cp258_1454		X		Cp258_1454	Cell wall/Membrane and Envelope biogenesis	Cellular processes and signaling	
I3QW80_CORPS	Resuscitation promoting factor RpfA	107514.1	0.92	0.64	0.05	1	0.748888	SEC	Cp258_0620		X		rpfA	Cell division and cellular cycle	Cellular processes and signaling	
I3QX17_CORPS	Unknown Function	50750.08	0.88	0.61	0.05	1	0.870145	SEC	Cp258_0912		X		Cp258_0912	Unknown Function	Poorly characterized	
I3QWB6_CORPS	Glutaredoxin	53685.86	0.79	0.55	0.13	1	0.916358	CYT	Cp258_0656				Cp258_0656	Post-translational modification, protein turnover, chaperones	Cellular processes and signaling	
I3R043_CORPS	ABC transporter substrate binding lipoprotein	1194.18	0.76	0.53	0.2	1	0.482534	PSE	Cp258_2022	X	X		yvrC	Intracellular trafficking, secretion, and vesicular transport	Cellular processes and signaling	
I3QYX1_CORPS	Peptide ABC transporter substrate-binding protein	2434.83	0.76	0.53	0.14	1	0.699664	PSE	Cp258_1574	X	X		oppA5	Intracellular trafficking, secretion, and vesicular transport	Cellular processes and signaling	
I3QUR9_CORPS	Unknown Function	3842.72	0.75	0.52	0.11	1	0.307009	PSE	Cp258_0080	X	X		Cp258_0080	Unknown Function	Poorly characterized	**>Cp258PiCp02**
I3QZM9_CORPS	Secretory lipase	30591.1	0.74	0.51	0.08	1	0.440754	SEC	Cp258_1848		X		lipY	General function prediction only	Poorly characterized	
I3QVF4_CORPS	Unknown Function	1359.85	0.68	0.47	0.31	1	0.960625	PSE	Cp258_0328		X		Cp258_0328	Unknown Function	Poorly characterized	
I3QYW5_CORPS	Iron ABC transporter ATP-binding protein	419.87	0.66	0.46	0.27	1	0.570171	PSE	Cp258_1567		X		Cp258_1567	Inorganic Ion Transport and Metabolism	Metabolism cellular	
I3QV73_CORPS	Hydrolase domain containing protein	70339.38	0.65	0.45	0.09	1	0.085063	SEC	Cp258_0246		X		Cp258_0246	General function prediction only	Poorly characterized	
I3QZB4_CORPS	DsbG protein	13965.91	0.62	0.43	0.1	1	0.853219	SEC	Cp258_1735		X		dsbG	Post-translational modification, protein turnover, chaperones	Cellular processes and signaling	**>Cp258PiCp14**
I3QY87_CORPS	SEC penicillin binding protein	2538.9	0.56	0.39	0.09	1	0.294749	SEC	Cp258_1330		X		pbpB	General function prediction only	Poorly characterized	
I3QWG4_CORPS	Resuscitation promoting factor RpfB	87332.26	0.53	0.37	0.03	1	0.706312	SEC	Cp258_0706		X		rpfB	Cell division and cellular cycle	Cellular processes and signaling	
I3QZ55_CORPS	L-D-transpeptidase YkuD	2127.53	0.49	0.34	0.18	1	0.925984	SEC	Cp258_1658		X		ykuD	Cell wall/Membrane and Envelope biogenesis	Cellular processes and signaling	
I3QUM7_CORPS	Phospholipase D	66863.88	0.49	0.34	0.04	1	0.510723	SEC	Cp258_0036		X		pld	Lipid transport and metabolism	Metabolism cellular	**>Cp258PiCp01**
I3QW39_CORPS	Unknown Function	2379.95	0.49	0.34	0.15	1	0.848798	PSE	Cp258_0579	X	X		Cp258_0579	Unknown Function	Poorly characterized	
I3QYG8_CORPS	Unknown Function	3374.89	0.48	0.33	0.13	1	0.793189	SEC	Cp258_1413		X		Cp258_1413	Unknown Function	Poorly characterized	
I3QXX8_CORPS	Copper resistance protein CopC	1251.92	0.45	0.31	0.25	0,98	0.964486	PSE	Cp258_1222		X		copC	Post-translational modification, protein turnover, chaperones	Cellular processes and signaling	
I3QV40_CORPS	Unknown Function	39773.54	0.45	0.31	0.06	1	0.921069	SEC	Cp258_0214		X	X	Cp258_0214	Unknown Function	Poorly characterized	**>Cp258PiCp13**
I3QZ73_CORPS	Unknown Function	23130.1	0.45	0.31	0.05	1	0.540475	SEC	Cp258_1678		X		Cp258_1678	Unknown Function	Poorly characterized	
I3QW57_CORPS	Unknown Function	97013.5	0.39	0.27	0.03	1	0.330983	SEC	Cp258_0596		X	X	Cp258_0596	Unknown Function	Poorly characterized	**>Cp258PiCp09**
I3QVF5_CORPS	ABC type metal ion transport system	1928.96	0.38	0.26	0.13	1	0.087456	PSE	Cp258_0329	X			Cp258_0329	Inorganic Ion Transport and Metabolism	Metabolism cellular	
I3QV79_CORPS	Surface layer protein A	162779.7	0.38	0.26	0.05	1	0.680647	SEC	Cp258_0252		X		slpA	General function prediction only	Poorly characterized	

I3QW45_CORPS	Copper containing nitrite reductase	641.59	0.33	0.23	0.14	1	0.893002	PSE	Cp258_0585				*aniA*	Post-translational modification, protein turnover, chaperones	Cellular processes and signaling	**>Cp258PiCp09**
I3QYY1_CORPS	Bacterial extracellular solute-binding proteins	3910.48	0.32	0.22	0.07	1	0.221793	PSE	Cp258_1585	X	X		*Cp258_1585*	General function prediction only	Poorly characterized	**>Cp258PiCp06**
I3QW47_CORPS	Unknown Function	1218.04	0.22	0.15	0.18	0,96	0.896967	SEC	Cp258_0587		X		*Cp258_0587*	Unknown Function	Poorly characterized	**>Cp258PiCp09**
I3QX38_CORPS	Cutinase	49567.47	0.14	0.1	0.04	1	0.528797	SEC	Cp258_0933		X		*Cp258_0933*	General function prediction only	Poorly characterized	
I3QZH4_CORPS	Corynomycolyl transferase	28335.13	-0.13	-0.09	0.03	0	0.621850	SEC	Cp258_1793		X		*Cp258_1793*	Cell wall/Membrane and Envelope biogenesis	Cellular processes and signaling	
I3QZS5_CORPS	Nitrite reductase periplasmic cytochrome c552	2075.56	-0.25	-0.17	0.15	0,04	0.880722	SEC	Cp258_1897		X		*nrfA*	Energy metabolism	Metabolism cellular	
I3QZ49_CORPS	Carbon starvation protein A	622.15	-0.43	-0.3	0.21	0,01	0.957095	MEM	Cp258_1651				*pcsA*	Defense mechanism	Cellular processes and signaling	**>Cp258PiCp10**
I3QW24_CORPS	Hydrolase domain containing protein	17234.12	-1.26	-0.87	0.06	0	0.493566	SEC	Cp258_0564		X		*Cp258_0564*	General function prediction only	Poorly characterized	
I3QWW3_CORPS	Diaminopimelate decarboxylase	600.78	-1.27	-0.88	0.32	0	0.049844	CYT	Cp258_0859				*lysA*	Amino acid transport and metabolism	Metabolism cellular	
I3QVZ1_CORPS	Unknown Function	2256.66	-1.28	-0.89	0.36	0	0.053598	CYT	Cp258_0531				*Cp258_0531*	Unknown Function	Poorly characterized	
I3QUW5_CORPS	Manganese zinc iron transport system ATP-binding	391.52	-1.38	-0.96	0.29	0	0.038111	CYT	Cp258_0127				*mntB*	Inorganic Ion Transport and Metabolism	Metabolism cellular	
I3QYP5_CORPS	MutT NUDIX family protein	5870.55	-1.38	-0.96	0.32	0	0.066920	CYT	Cp258_1498				*Cp258_1498*	General function prediction only	Poorly characterized	
I3QW96_CORPS	Enoyl CoA hydratase echA6	611.75	-1.4	-0.97	0.22	0	0.037919	CYT	Cp258_0636				*echA6*	Lipid transport and metabolism	Metabolism cellular	
I3QYV3_CORPS	Unknown Function	141.46	-1.53	-1.06	0.28	0	0.032554	CYT	Cp258_1555				*Cp258_1555*	Unknown Function	Poorly characterized	
I3QXT1_CORPS	Chorismate synthase aroC	614.35	-1.54	-1.07	0.4	0	0.057804	CYT	Cp258_1174				*aroC*	Amino acid transport and metabolism	Metabolism cellular	
I3QV42_CORPS	Protein yqeY	23153.72	-1.57	-1.09	0.48	0	0.038791	CYT	Cp258_0216				*yqeY*	General function prediction only	Poorly characterized	
I3R0F7_CORPS	Anthranilate synthase component II	382.53	-1.63	-1.13	0.47	0	0.064911	CYT	Cp258_2136				*trpG*	General function prediction only	Poorly characterized	
I3QY54_CORPS	4-hydroxy-tetrahydrodipicolinate reductase	1212.64	-1.67	-1.16	0.26	0	0.044599	CYT	Cp258_1298				*dapB*	Amino acid transport and metabolism	Metabolism cellular	
I3QWK1_CORPS	Unknown Function	221.77	-2.02	-1.4	0.43	0	0.968462	MEM	Cp258_0745				*Cp258_0745*	Unknown Function	Poorly characterized	
I3R080_CORPS	Unknown Function	2564.2	-2.64	-1.83	0.27	0	0.535121	PSE	Cp258_2060	X	X	X	*Cp258_2060*	Unknown Function	Poorly characterized	
I3QXJ1_CORPS	Prolipoprotein LppL	2671.28	-3.38	-2.34	0.15	0	0.626055	PSE	Cp258_1084	X	X		*lppL*	General function prediction only	Poorly characterized	
I3QX04_CORPS	Mycothiol acetyltransferase	5934.73	-4.62	-3.2	0.37	0	0.278762	CYT	Cp258_0899				*mshD*	Cell wall/Membrane and Envelope biogenesis	Cellular processes and signaling	
I3QZA3_CORPS	Protein NrdI	7211.97	-4.7	-3.26	0.22	0	0.074033	CYT	Cp258_1723				*nrdI*	General function prediction only	Poorly characterized	

SecP value = SecP score, value above 0.5 indicates possible secretion

PIPs = pathogenicity islands prediction (Soares et al., 2012)

(A) Ratio values to: Rc:Ct_Log(2) Ratio > 1.2, p > 0.95 = up-regulation, p < 0.05 = down-regulation
