# Supplementary material for: Quantitative Proteomic Analysis Reveals Changes in the Benchmark Corynebacterium pseudotuberculosis Biovar Equi Exoproteome after Passage in a Murine Host
Source: Front Cell Infect Microbiol. 2017 Jul 25;7:325. doi: 10.3389/fcimb.2017.00325 (PMC5524672; doi:10.3389/fcimb.2017.00325)
Supplement: Supplementary Table 3 — Proteins unique to the recovered and control conditions. [file Table3.PDF]

Supplementary Table S.3 = Proteins unique to the recovered and control conditions.

Accession	Description	Score	Condition	Biological Process	Biological Process	SignalP	LipoP	Gene symbol	Subcellular localization	SecP
I3QZS6_CORPS	Cytochrome c nitrate reductase small subunit NrfH	1035.81	Control	Energy metabolism	Metabolism cellular			*nfrC*	MEM	0.898422
I3QXN4_CORPS	Cytochrome oxidase assembly protein	893.51	Recovered	Energy metabolism	Metabolism cellular			*Cp258_1127*	MEM	0.966085
I3QWA4_CORPS	Multidrug resistance protein norM	561.85	Recovered	Defense mechanism	Cellular processes and signaling			*norM*	MEM	0.968227
I3QY62_CORPS	Multidrug resistance protein norM	487.65	Recovered	Defense mechanism	Cellular processes and signaling			*norM*	MEM	0.971397
I3QV51_CORPS	Thioredoxin related protein	259.85	Recovered	Post-translational modification, protein turnover, chaperones	Cellular processes and signaling	X		*Cp258_0224*	SEC	0.623525
I3QVD0_CORPS	Unknow function	836.52	Recovered	Unknow function	Poorly characterized	X		*Cp258_0305*	SEC	0.951009
I3QVQ7_CORPS	Unknow function	3502.56	Recovered	Unknow function	Poorly characterized	X		*Cp258_0448*	SEC	0.628003
I3QWR5_CORPS	Unknow function	1286.94	Recovered	Unknow function	Poorly characterized	X		*Cp258_0810*	SEC	0.811667

SecP value = SecP score, value above 0.5 indicates possible secretion
